# EMDR as Add-On Treatment for Psychiatric and Traumatic Symptoms in Patients with Substance Use Disorder

**DOI:** 10.3389/fpsyg.2017.02333

**Published:** 2018-01-11

**Authors:** Sara Carletto, Francesco Oliva, Micaela Barnato, Teresa Antonelli, Antonina Cardia, Paolo Mazzaferro, Carolina Raho, Luca Ostacoli, Isabel Fernandez, Marco Pagani

**Affiliations:** ^1^Clinical and Biological Sciences Department, University of Turin, Orbassano, Italy; ^2^EMDR Italy Association, Bovisio Masciago, Italy; ^3^Associazione l'Arcobaleno, Comunità di Capodarco di Fermo, Fermo, Italy; ^4^Ser.T, Limbiate, Italy; ^5^Clinical and Oncological Psychology, Città della Salute e della Scienza Hospital of Turin, Turin, Italy; ^6^Institute of Cognitive Sciences and Technologies, National Research Council (CNR), Rome, Italy

**Keywords:** eye movement desensitization and reprocessing, substance use disorder, traumatic stress, dissociation, anxiety, depression, psychiatric symptoms, adverse childhood experiences

## Abstract

**Background:** Substance use disorders (SUD) are patterns of substance use leading to severe impairment on social, working and economic levels. *In vivo* and clinical findings have enhanced the role of the brain's stress-related system in maintaining SUD behaviors. Several studies have also revealed a high prevalence of post-traumatic symptoms among SUD patients, suggesting that a trauma-informed treatment approach could lead to better treatment outcomes. However, only few studies have evaluated the use of eye movement desensitization and reprocessing (EMDR) in SUD without consistent results. The aim of the present pilot study was to assess the efficacy of a combined trauma-focused (TF) and addiction-focused (AF) EMDR intervention in treating post-traumatic and stress-related symptoms of patients with SUD.

**Methods:** Forty patients with different SUD were enrolled in the study. Twenty patients underwent treatment as usual (TAU), the other 20 patients were treated with TAU plus 24 weekly sessions of EMDR. All patients were assessed before and after intervention for several psychological dimensions using specific tools (i.e., BDI-II, DES, IES-R, STAI, and SCL-90-GSI). A repeated measure MANOVA was performed to evaluate both between groups (TAU + EMDR vs. TAU) and within group (pre- vs. post-intervention) effects and interactions. A secondary outcome was the dichotomous variable yielded by the urine drug testing immunoassay (yes/no).

**Results:** The RM-MANOVA revealed both a significant pre–post main effect (*p* < 0.001), and a significant group-by-time main effect (*p* < 0.001). Significant improvements on IES-R, DES, and SCL-90-GSI scales were shown in both groups according to time effects (*p* < 0.05). However, significant greater effects were found for TAU + EMDR group than TAU group. No differences were found between TAU and TAU + EMDR groups in terms of urine drug immunoassay results before and after the interventions.

**Conclusions:** The TAU + EMDR group showed a significant improvement of post-traumatic and dissociative symptoms, accompanied by a reduction in anxiety and overall psychopathology levels, whereas TAU group showed a significant reduction only in post-traumatic symptoms. Although our results can only be considered preliminary, this study suggests that a combined TF- and AF- EMDR protocol is an effective and well-accepted add-on treatment for patients with SUD.

## Introduction

Substance use disorders (SUD) are pathological patterns of behaviors related to substance use leading to severe impairment of familial, social and working relationships as well as of economic conditions (American Psychiatric Association, 2013).

Although the neurobiological circuitry that is associated with drug reward has been broadened in recent years, the meso-cortical-striatal dopamine system is still the most important pathway involved in the rewarding properties of almost all drugs (Koob and Volkow, 2016).

However, *in vivo* and clinical findings have also enhanced the role of brain's stress-related system in maintaining SUD behaviors: the chronic administration of all major drugs with dependence or abuse potential is associated with corticotropin-releasing factor variation leading to both hypothalamic-pituitary-adrenal axis and brain stress system dysregulation (Koob, 2013).

The increase of corticotropin-releasing factor, dynorphin, and norepinephrine recruited in the extended amygdala contributes to the development of negative emotional states during acute withdrawal (such as chronic irritability, dysphoria, and loss of motivation; Koob and Volkow, 2016).

From an epidemiologic point of view, patients having any lifetime SUD showed higher risk of also having a post-traumatic stress disorder (PTSD; OR = 1.6, 95% CI = 1.27–2.10, Grant et al., 2016) with a prevalence of current PTSD ranging from 15 to 42% (Mills et al., 2005; Reynolds et al., 2005, 2011; Driessen et al., 2008).

Moreover, some studies conducted on SUD showed that 67–92% of the patients report having experienced at least one traumatic event according to the DSM-IV PTSD criterion A (Dragan and Lis-Turlejska, 2007; Reynolds et al., 2011).

Furthermore, several studies have also reported a strong relationship between exposure to severe stress in childhood and substance abuse (Dube et al., 2003; Green et al., 2010). One of the most important studies, conducted by the Center for Disease Control along with the Kaiser Hospital in San Diego, released the landmark Adverse Childhood Experience (ACE) study, showing that individuals who experienced four or more types of ACEs were at a four to 12-fold increased risk of developing alcohol or drug abuse problems (Felitti et al., 1998).

Research has shown that substance abuse treatment using a trauma-informed approach could lead to better treatment outcomes, such as greater symptom reduction and increased retention in treatment (Amaro et al., 2007; LeTendre and Reed, 2017).

Such involvement of stress systems, trauma, and PTSD in SUD suggested a possible role of intervention possibly impacting on traumatic and stress disorders in the treatment of patients with SUD.

Among the different psychological approaches, eye movement desensitization and reprocessing (EMDR) has emerged as an evidence-based therapy for the treatment of psychological sequelae of traumatic events and other negative stressful experiences (Shapiro, 2014).

EMDR is a psychotherapeutic approach that focuses on trauma elaboration. It is guided by the adaptive information processing (AIP) model, that posits that stressful events not fully processed and integrated into the already existing memory networks are stored in a dysfunctional way. A distinct characteristic of EMDR therapy is the use of alternating bilateral stimulation (eye movements, tactile, or audio), which appears to produce a physiological effect promoting accelerated reprocessing of dysfunctionally stored information related to the traumatic event (Jeffries and Davis, 2013; Carletto et al., 2017; Pagani et al., 2017).

EMDR is considered one of the elective psychotherapeutic treatments for PTSD, according to several meta-analyses and clinical guidelines (Van Etten and Taylor, 1998; Davidson and Parker, 2001; Bradley et al., 2005; National Collaborating Centre for Mental Health, 2005; Bisson et al., 2013; WHO, 2013; Chen et al., 2014, 2015) and its neurobiological effects are also supported by neuroimaging findings (Pagani et al., 2012, 2015; Boukezzi et al., 2017).

Furthermore, in recent years the use of EMDR has expanded beyond PTSD and several studies have reported its efficacy for treatment of trauma-associated symptoms in patients with other psychiatric conditions (for a review see Valiente-Gómez et al., 2017). Among these, several protocols of treatment were developed in order to address traumatic experiences of SUD patients.

The clinical application of trauma-focused EMDR (TF-EMDR) in some studies resulted in EMDR being efficacious in the treatment of traumatic symptoms, but not in addiction behavior severity (see reviews by Roberts et al., 2015 and Markus and Hornsveld, 2017). Subsequently, some authors focused on the role of TF-EMDR in patients with SUD without PTSD, considering different types of outcomes even in relation to the addiction with fairly positive results but without conclusive findings.

Finally, as a third possible application of EMDR in SUD, there were some proposals of addiction-focused EMDR (AF-EMDR) protocols, such as the desensitization of triggers and urge reprocessing (DeTUR) protocol by Popky (2005), the feeling-state addiction protocol (FSAP) by Miller (2010) and the craving extinguished (CravEx) protocol by Hase et al. (2008). All these protocols were specifically focused on the addiction rather on trauma but only the CravEx was clinically evaluated in a randomized clinical trial. Comparing treatment as usual (TAU) with CravEx plus TAU in a sample of patients with alcohol use disorder, Hase et al. (2008) have found a significant reduction in craving and depression severity up to 1 month after treatment.

To the best of our knowledge, no studies have yet evaluated the efficacy of both trauma and addiction-focused protocols on the relapse rate and stress-related symptoms of patients with SUD. Therefore, the aim of the present pilot study was to assess the efficacy of a combined trauma-focused and addiction-focused EMDR protocol in treating post-traumatic and stress-related symptoms of patients with SUD. We hypothesized that this combined adjunctive EMDR intervention would be more effective than a TAU intervention.

## Materials and methods

### Design

This was a quasi-experimental study investigating the efficacy of an additional EMDR treatment as compared with TAU alone in patients diagnosed with SUD.

### Setting

The participants were recruited in two settings: an outpatient territorial service for drug addiction in northern Italy (Ser.T. of Limbiate, MI) and a residential facility in central Italy (Comunità di Capodarco di Fermo, FM) from March 2015 to May 2016.

The study was approved by the Medical Ethics Committee of Azienda Territoriale dei Servizi of Brianza (MB, Italy) and by the Board of Directors of Capodarco (FM, Italy). Informed written consent was obtained from all the participants.

### Participants

The subjects of the study were patients with a diagnosis of SUD, who were referred to one of the two above-mentioned centers for drug addiction treatment.

Inclusion criteria were as follows: (1) a diagnosis of SUD, according to DSM-5; (2) age between 18 and 65 years; (3) fluent Italian language; (4) legal capacity to consent to the treatment; (5) maintenance of psychotropic medications throughout the study.

Exclusion criteria were as follows: (1) having a pathological gambling disorder without comorbidity with other SUDs; (2) presence of other severe psychiatric disorders such as psychosis or bipolar disorder; (3) cognitive disorders such as overt dementia; (4) suicide attempts; (5) current pregnancy.

### Assessment

The recruitment of participants was carried out by a psychiatrist and psychologist who proposed participation in the research protocol to patients during a clinical visit in the outpatient setting and during the first visit after admission in the inpatient setting. The research protocol was proposed to consecutive patients who met the inclusion criteria, with an explanation of the aims of the study, and patients were asked whether they were willing to receive an additional psychotherapeutic intervention (EMDR) other than TAU. Patients could choose the group to which they wanted to be assigned (TAU or TAU + EMDR). On reaching the maximum number of patients in the TAU + EMDR group, the remaining patients were assigned to the TAU alone group.

The psychological assessment was performed by psychologists independent of the research protocol, using the same timing and tools, i.e., at baseline before the first session of treatment (T0), and after the end of treatment (T1).

The following psychological self-report questionnaires were administered:

*Impact of Event Scale—Revised (IES-R)*. The IES-R (Weiss and Marmar, 1997) is a 22- item self-report questionnaire consisting of three subscales (eight items relate to intrusions, eight items evaluate avoidance, and six items assess hyperarousal). The scale assesses subjective distress caused by traumatic events. An IES-R score equal to or >33 represents the best cut-off for a probable diagnosis of PTSD. The IES-R was found to be highly internally consistent (Cronbach's alpha, α = 0.96; Creamer et al., 2003).

*State-Trait Anxiety Inventory (STAI-Y)*. The STAI-Y (Spielberger et al., 1983) is used to measure the presence and severity of current symptoms of anxiety (state anxiety; STAI-1) and a generalized propensity to be anxious (trait anxiety; STAI-2). Range of scores for each subtest is 20–80, the higher score indicating greater anxiety. A cutoff point of 39–40 has been suggested to detect clinically significant symptoms for the state anxiety scale. The STAI-Y has shown an adequate to excellent internal reliability (α = 0.86–0.95).

*Beck Depression Inventory-II (BDI-II)*. The BDI-II (Beck and Steer, 1993) is a 21-item self-report instrument that assesses the presence and severity of depression symptoms. A score above 13 indicates presence of depression symptoms. The internal consistency of the BDI-II is good to excellent (α = 0.83–0.96; Wang and Gorenstein, 2013).

*Symptom Checklist 90 Items revised version (SCL-90- R)* (Derogatis et al., 1973; Derogatis, 1994) is a 90-items self-report questionnaire that evaluates a broad range of psychological problems and symptoms of psychopathology. For the purpose of this study we chose to utilize the Global Severity Index (GSI), as it represents the best global indicator of the intensity of psychic distress reported by the subject and it demonstrated a high Cronbach's alpha value (α = 0.97; Prinz et al., 2013). This global index combines information about the number of reported symptoms and the intensity of perceived discomfort. A score between 55 and 65 indicates a distress level of moderate intensity, while a score above 65 reveals a severe intensity of discomfort, beyond the threshold of clinical attention.

*Dissociative Experiences Scale (DES)* (Bernstein and Putnam, 1986; Frischholz et al., 1990) is a brief, 28-item, self-report inventory of the frequency of dissociative experiences. It represents a reliable and valid measure for determining the contribution of dissociation to various psychiatric disorders and a screening instrument for dissociative disorders. High levels of dissociation are indicated by scores of 30 or more. The DES has an excellent internal consistency, with Cronbach's alpha ranging from 0.96 to 0.97 (Dubester and Braun, 1995).

The *Adverse Childhood Experience Questionnaire (ACE)* (Felitti et al., 1998) is a 10-item self-report measure developed for the ACE study to identify childhood experiences of abuse and neglect. The internal consistency of the ACE questionnaire is adequate (α = 0.88; Murphy et al., 2014). This questionnaire was administered only at baseline.

### Treatments

#### Treatment as usual

All patients received TAU, which consisted of standard treatment for recovery from SUD in the National Health Service in Italy. TAU included clinical interviews with the addiction specialist and administration of medications appropriate for each patient (e.g., alcohol craving, heroin substitute treatment). Comorbid psychiatric conditions such as depression or anxiety disorders were treated in accordance with the patient's needs, including appropriate medication.

Lastly, TAU included psychological treatment (both individual and group sessions) and participation in psycho-educational group sessions.

#### Eye movement desensitization and reprocessing

Participants received 24 weekly EMDR sessions over a period of 6 months. The EMDR treatment used in this study incorporated both elements of the classic TF-EMDR protocol (Shapiro, 2001) and of the existing AF-EMDR protocols (Hase, 2010; Knipe, 2010; Miller, 2010; Popky, 2010), in accordance with the Palette of EMDR Interventions in Addiction (PEIA; Markus and Hornsveld, 2017).

The EMDR treatment steps were as follows:

1. Building a positive therapeutic relationship;
2. Information gathering (trauma history, addiction history);
3. Strengthening the motivation for treatment through positive and achievable therapeutic goals and enhancing personal resources;
4. Desensitization of traumatic events in chronological order;
5. Desensitization of the “first time” memory and the dependence of precipitating factors;
6. Desensitization of the level of urge;
7. Desensitization of the recall of the relapse;
8. Desensitizing triggers of triggering behavior;
9. Installing a positive state for each triggering factor.

EMDR treatment was provided by four clinical psychotherapists specialized in EMDR therapy (who at least had completed the Level II EMDR program). The EMDR therapists were supervised monthly by an EMDR consultant.

### Statistical analyses

Data were processed and analyzed using the Statistical Package for Social Sciences (SPSS version 22.0; Chicago, IL, USA).

Both parametric and nonparametric tests were used, in accordance with Shapiro–Wilk as a test for normality. Baseline group differences were assessed using Student's *t*-test or Mann–Whitney *U*-test to compare the two groups for continuous measures and Fisher's Exact Test for categorical measures.

GLM repeated measures multivariate ANOVA (RM-MANOVA) was used to analyze the main pre- and post- intervention effects and interactions both between and within TAU + EMDR and TAU groups. Pairwise comparison between groups were made by simple contrast and are reported as means difference with the Sidak correction 95% confidence interval (95%CI) for multiple comparisons.

A *p* < 0.05 was considered statistically significant throughout all of the analyses.

## Results

A total of 40 patients were enrolled in the study: 20 were assigned to the TAU + EMDR intervention and the other 20 patients were assigned to the TAU treatment. We did not register any dropout from the treatments.

Table 1 presents the sociodemographic characteristics of these patients at baseline. There were no significant differences in demographics between the two groups at baseline (T0), except for adverse childhood experiences, which were more frequent in the TAU + EMDR group (Table 1).

**Table 1:** Demographic data of participants at baseline.

	**EMDR (*N* = 20) Mean (*SD*)/Median (IQR)**	**TAU (*N* = 20) Mean (*SD*)/Median (IQR)**	***p***
Age (years)	32 (8)	32 (19)	0.820^a^
Years of substance use	19.40 (7.98)	21.10 (9.59)	0.546^b^
Adverse Childhood Experiences	4 (5)	2 (2)	0.004^a^
	***n*** **(%)**	***n*** **(%)**	
Gender			0.487^c^
Female	2 (10)	0 (0)	
Male	18 (90)	20 (100)	
Marital status			0.410^c^
Single	17 (85)	14 (70)	
Married	1 (5)	4 (20)	
Separated/divorced	2 (10)	2 (10)	
Level of education			0.198^c^
Primary school	0 (0)	3 (15)	
Low secondary school	9 (45)	10 (50)	
High secondary school	11 (55)	7 (35)	

*EMDR, Eye Movement Desensitization and Reprocessing group; TAU, Therapy As Usual group*.

a *Mann–Whitney U-test*.

b *Pearson's independent samples t-test*.

c *Fisher's exact test*.

There were several differences between the two groups at baseline. Overall, patients in the TAU + EMDR group showed higher post-traumatic stress and anxiety symptoms and more psychiatric symptoms.

We evaluated whether the different psychotherapy treatments (TAU + EMDR or TAU) administered to the patients had a different impact on the psychological variables of interests. A repeated-measures MANOVA was performed on the pre- and post-intervention clinical scores (IES-R, DES, SCL-90-GSI, STAI-1, STAI-2, BDI-II), comparing group and time effects and interactions between group and time.

The RM-MANOVA yielded a significant pre–post main effect [*F*_(6, 33)_ = 10.102, *p* < 0.001; η^2^_*p*_ = 0.647], and a significant interaction between the pre–post measures and the treatment condition [*F*_(6, 33)_ = 7.830, *p* < 0.001; η^2^_*p*_ = 0.587].

Significant time effects were found across both groups for all variables except for STAI-1 and STAI-2, indicating that the mean participant scores improved from time 0 (pre-intervention) to time 1 (post-intervention) on all variables except for anxiety symptoms (Table 2).

**Table 2:** Comparison of clinical variables for the two groups (TAU and TAU + EMDR).

	**Pre-treatment**		***p***	**Post-treatment**		***p***	**Effect Time**			**Effect Time × Group**		
	**TAU (*N* = 20)**	**TAU + EMDR (*N* = 20)**		**TAU (*N* = 20)**	**TAU + EMDR (*N* = 20)**		***F***	***P***	**η^2^*_*p*_***	***F***	***P***	**η^2^*_*p*_***
BDI-II	11.60 (7.45)	18.35 (14.08)	0.066	10.10 (7.58)	11.65 (12.54)	0.639	8.646	0.006	0.185	3.477	0.070	0.084
STAI-1	41.95 (4.17)	46.35 (5.26)	0.006	46.25 (5.28)	43.50 (5.31)	0.109	0.459	0.502	0.012	11.160	0.002	0.227
STAI-2	42.05 (2.69)	45.65 (5.49)	0.012	43.20 (3.14)	42.60 (7.61)	0.746	1.476	0.232	0.037	7.212	0.011	0.160
DES	10.93 (8.07)	15.69 (14.05)	0.196	8.53 (6.67)	6.72 (7.13)	0.411	15.766	<0.001	0.293	5.279	0.027	0.122
IES-R	23.90 (15.35)	39.65 (23.12)	0.015	12.30 (11.76)	6.05 (5.88)	0.040	48.282	<0.001	0.560	11.438	0.002	0.231
SCL-90-GSI	62.65 (10.39)	73.90 (2.94)	<0.001	61.95 (11.55)	63.25 (12.37)	0.733	14.378	0.001	0.275	11.050	0.002	0.225

*Data are mean (SD)*.

TAU, Therapy As Usual group;

*TAU + EMDR, Eye Movement Desensitization and Reprocessing in addition to TAU group*.

Group-by-time interaction effects were found for IES-R, DES, SCL-90-GSI, STAI-1, and STAI-2 total scores, indicating that clinical improvements regarding these variables were different in the two treatment groups. No group-by-time interaction was found for BDI-II, showing that change on this measure was similar for both treatment groups (Table 2).

Planned *post-hoc* analyses of simple effects of pre–post were conducted for all variables with a significant group-by-time effect (DES, IES-R, SCL-90-GSI, STAI-1, STAI-2,) by GLM pairwise comparisons using the Sidak adjustment for multiple comparisons.

The two groups significantly differ for IES-R scores at baseline, with participants in the TAU + EMDR group showing higher post-traumatic symptoms than those in the TAU group (Table 2). The analysis of simple effects over time indicated both groups had an improvement in post-traumatic symptoms (Table 3), but the TAU + EMDR group scored significantly lower compared to the TAU group at post-treatment (Table 2).

**Table 3:** Comparison between T0 and T1 of clinical variables for the two groups (TAU and TAU + EMDR).

	**TAU**				**TAU + EMDR**			
	**T0**	**T1**	**Mean difference (95%CI)**	***p***	**T0**	**T1**	**Mean difference (95%CI)**	***p***
BDI-II	11.60 (7.45)	10.10 (7.58)	−1.500 (−5.492; 2.492)	0.452	18.35 (14.08)	11.65 (12.54)	−6.700 (−10.692; −2.708)	0.002
STAI-1	41.95 (4.17)	46.25 (5.28)	4.300 (1.236; 7.384)	0.007	46.35 (5.26)	43.50 (5.31)	−2.850 (−5.914; 0.214)	0.067
STAI-2	42.05 (2.69)	43.20 (3.14)	1.150 (−1.089; 3.389)	0.305	45.65 (5.49)	42.60 (7.61)	−3.050 (−5.289; −0.811)	0.009
DES	10.93 (8.07)	8.53 (6.67)	−2.395 (−6.493; 1.703)	0.244	15.69 (14.05)	6.72 (7.13)	−8.973 (−13.071; −4.874)	<0.001
IES-R-Total	23.90 (15.35)	12.30 (11.76)	−11.600 (−20.912; −2.288)	0.016	39.65 (23.12)	6.05 (5.88)	−33.600 (−42.912; −24.288)	<0.001
SCL-90 Total	62.65 (10.39)	61.95 (11.55)	−0.700 (−4.985; 3.585)	0.743	73.90 (2.94)	63.25 (12.37)	−10.650 (−14.935; −6.365)	<0.001

*Data are mean (SD)*.

TAU, Therapy As Usual group;

*TAU + EMDR, Eye Movement Desensitization and Reprocessing in addition to TAU group*.

As regards the DES score, there was no significant difference between groups at baseline (Table 2). Results indicated that the group-by-time effect is explained by the significant difference between dissociative pre- and post-treatment scores for participants who underwent EMDR intervention (Table 3).

Moreover, there was also a difference between groups at baseline for the SCL-90-GSI score, with more severe psychiatric symptoms in the TAU + EMDR group (Table 2). The comparison between pre- and post-treatment indicated a significant improvement in the TAU + EMDR group between T0 and T1, while there was no difference in the TAU group (Table 3).

In the case of STAI-1, results indicated that there was a significant difference between the two groups at baseline, as the STAI-1 scores at baseline in TAU + EMDR group were significantly higher than those in TAU group (Table 2). Concurrently, there was a significant difference between STAI-1 pre- and post-treatment scores in the TAU group but not in the TAU + EMDR group. This indicates that the group-by-time effect was due to the significant difference between groups at baseline and to the significant worsening of state anxiety symptoms in patients in the TAU group (Table 3).

With regard to STAI-2, a significant difference between the two groups at baseline was found, as STAI-2 scores at baseline in TAU + EMDR group were significantly higher than those in TAU group (Table 2). Moreover, there was a significant reduction of STAI-2 scores in the TAU + EMDR group that was not present in the TAU group. This indicates that the improvements over time on trait anxiety were registered only in the TAU+ EMDR treatment group (Table 3).

No differences were found before and after treatment in the urine drug testing immunoassays, which showed a similar increase of negative results after the interventions (TAU group from 65% at baseline to 85% at T1; TAU + EMDR group from 70% at baseline to 80% at T1; χ^2^ = 0.067, *p* = 0.795).

## Discussion

Overall, all SUD patients included in the study improved their clinical condition with a significant reduction of post-traumatic, dissociative and psychiatric symptoms, regardless of the type of treatment.

Both TAU and TAU + EMDR interventions had a significant effect in reducing post-traumatic symptoms, but the add-on EMDR proved to have a significant greater effect, allowing a shift from baseline levels above the clinical cut-off to post-treatment normal levels. This finding is in line with those of previous studies (Perez-Dandieu and Tapia, 2014; Brown et al., 2015), which showed that adding EMDR to TAU has a significant effect on post-traumatic symptoms.

In the same way, according to the results of the present study, the add-on EMDR has an important effect in reducing dissociative symptoms, probably due to the well-recognized effect of EMDR on the reintegration of previous dysfunctionally stored memories (Nardo et al., 2013; van der Hart et al., 2013).

As regards the effect of EMDR on stress-related psychiatric symptoms, a significant improvement in the global severity of psychiatric symptoms was observed in patients who received add-on EMDR as compared to TAU alone, suggesting that EMDR also has a beneficial impact on a wide range of symptoms of clinical relevance, beyond post-traumatic symptoms.

In terms of anxiety, our results show a significant effect of add-on EMDR in improving trait anxiety that is not shown in TAU alone. In spite of its tendency to be stable over time, a number of studies revealed that trait anxiety can improve as a result of a psychological intervention over time (Vøllestad et al., 2011; Lee et al., 2015). Our results suggest that EMDR intervention might also affect the trait-like tendency to experience anxiety over time and across situations. Another interesting finding of our study is that state anxiety worsened in the TAU alone group, whereas in the TAU + EMDR group it remained stable. An increase of anxiety levels, mediated by adrenocorticotropic hormone, corticosterone, and amygdala corticotrophin releasing factor (CRF), is commonly observed during acute withdrawal stages of substance treatment and recovery programs (Koob and Volkow, 2016). It would seem that the TAU alone does not impact on this increase in anxiety levels, whereas the add-on of an EMDR intervention seems to be able to counterbalance this physiological elevation of anxiety related to abstinence.

With regard to depressive symptoms, no significant change was observed in either group, although our findings suggest a trend toward improvement in the group that received add-on EMDR, partially confirming previous findings (Hase et al., 2008; Perez-Dandieu and Tapia, 2014).

This study presents a methodological limitation that may moderate the interpretation of the results outlined so far. The non-randomized design led to the significant differences between the two groups at baseline. In fact, participants who received EMDR treatment showed higher baseline levels of symptoms compared to the group receiving only TAU treatment. These differences at baseline could limit a conclusive interpretation of the results of the study, as the improvements obtained by the group that received EMDR in addition to TAU could also be due to a spontaneous reduction of symptoms linked to the fact that higher reductions are observed when there are higher starting levels.

At the same time, the findings of the present study suggest that EMDR may be more useful in subjects who experienced more adverse childhood experiences and higher levels of symptoms, in order to strengthen standard treatment that otherwise would only be partially effective, especially on withdrawal-related anxiety. Consistent with previous literature reporting that adverse childhood events have significant implications for substance abuse treatment and that a trauma-informed approach to SUD leads to better treatment outcomes (Felitti et al., 1998; LeTendre and Reed, 2017), our findings suggest that exposure to adverse childhood experiences should be routinely assessed in treatment settings, in order to provide specific interventions to reduce traumatic burden associated with SUD. Future randomized controlled studies with larger samples should better investigate these aspects.

Another limit of the present study is that aspects related to craving and abstinence were not specifically investigated. The results of our study are in line with previous studies, which show that EMDR has beneficial effects on symptoms related to the traumatic history and only limited effects on additional outcomes (Markus and Hornsveld, 2017). The present study aimed to focus on post-traumatic and associated aspects linked to the relationship between addiction and traumatic burden, but future studies on similar populations should also take into account addict-related aspects.

This study also has some strengths. The results of the study confirm that EMDR could be a viable and well-accepted add-on treatment for patients with SUD, with some evidence of both efficacy and good compliance. Moreover, to the best of our knowledge this is the first study evaluating the clinical impact of an add-on EMDR intervention focused on both traumatic and addiction-related memories, and it found the first promising evidence of the efficacy of this combined TF- and AF-EMDR protocol. Further studies could evaluate the usefulness of combining TF- and AF-EMDR protocols in different clinical samples.

Although our results can only be considered preliminary, this study suggests that add-on EMDR is more effective than TAU alone in improving post-traumatic and dissociative symptoms, accompanied also by a reduction in anxiety and overall psychopathology levels.

The findings of this study underline the importance of assessing ACEs and other traumatic experiences in this population because they may contribute to the onset and maintenance of SUDs and lead to a worsening of psychopathological severity. As a clinical consequence, it could be useful to offer these patients specific add-on treatments addressing both ACEs and traumatic experiences related to addiction, in adjunction to standard treatments.

Future studies, such as that designed by Markus et al. (2015) on alcohol-dependent patients, would be better to investigate not only the effectiveness of an EMDR add-on treatment but also the mediators, moderators, and predictors of treatment outcome, in order to be able to delineate effective interventions for these disorders, which represent a major public health problem.

## Author contributions

MB is the national coordinator of the research. MB, IF, and MP were responsible for the conception and the design of the study. MB, TA, AC, PM, CR, and IF were responsible for data collection and for clinical treatments. SC and FO were responsible for the data analysis. IF, MB, LO, and MP contributed to the interpretation of data. SC and FO wrote the article, which was critically revised by all the others authors. All authors have approved the final version of the manuscript.

### Conflict of interest statement

IF is the president of EMDR Europe Association and the president of EMDR Italy Association. SC, LO, and MP have been invited speakers in national and international EMDR conferences. The other authors declare that the research was conducted in the absence of any commercial or financial relationships that could be construed as a potential conflict of interest. The handling Editor declared a shared affiliation, though no other collaboration, with several of the authors, SC, FO, and LO, and states that the process nevertheless met the standards of a fair and objective review.
